# Interaction of the Hippo Pathway and Phosphatases in Tumorigenesis

**DOI:** 10.3390/cancers12092438

**Published:** 2020-08-27

**Authors:** Sahar Sarmasti Emami, Derek Zhang, Xiaolong Yang

**Affiliations:** Department of Pathology and Molecular Medicine, Queen’s University, Kingston, ON K7L 3N6, Canada; saharsarmasti1990@gmail.com (S.S.E.); 16dyz@queensu.ca (D.Z.)

**Keywords:** Hippo pathway, protein phosphatase, tumorigenesis, YAP, TAZ, LATS, MST

## Abstract

**Simple Summary:**

Recently, a group of genes called “Hippo” has been discovered that is critical for the development and progression of a wide types of cancers. Therefore, modulating the “Hippo” activity is one of the most important ways to stop cancer. In this review, we have summarized for the first-time recent research findings on the crosstalk between “Hippo” and another group of genes called phosphatases. We have also proposed future directions of this research field. Our review provides very useful information on targeting of Hippo-phosphatases interactions for more effective cancer therapies in the future.

**Abstract:**

The Hippo pathway is an emerging tumor suppressor signaling pathway involved in a wide range of cellular processes. Dysregulation of different components of the Hippo signaling pathway is associated with a number of diseases including cancer. Therefore, identification of the Hippo pathway regulators and the underlying mechanism of its regulation may be useful to uncover new therapeutics for cancer therapy. The Hippo signaling pathway includes a set of kinases that phosphorylate different proteins in order to phosphorylate and inactivate its main downstream effectors, YAP and TAZ. Thus, modulating phosphorylation and dephosphorylation of the Hippo components by kinases and phosphatases play critical roles in the regulation of the signaling pathway. While information regarding kinase regulation of the Hippo pathway is abundant, the role of phosphatases in regulating this pathway is just beginning to be understood. In this review, we summarize the most recent reports on the interaction of phosphatases and the Hippo pathway in tumorigenesis. We have also introduced challenges in clarifying the role of phosphatases in the Hippo pathway and future direction of crosstalk between phosphatases and the Hippo pathway.

## 1. Introduction

### 1.1. The Hippo Pathway in Tumorigenesis and Its Regulation by Phosphorylation

**Figure 1:**
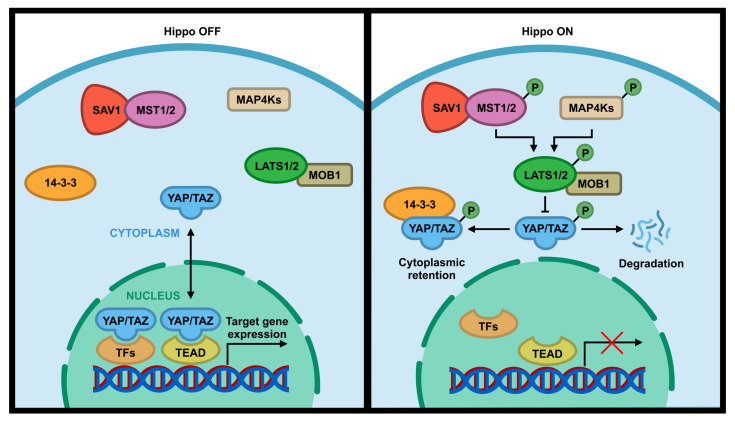
Core components of the Hippo pathway in mammalian cells. When the Hippo pathway is ON, MST1/2 and MAP4K are activated, which subsequently phosphorylate and activate LATS1/2 kinases. Activated LATS1/2 phosphorylate transcriptional coactivator YAP/TAZ, preventing entry into the nucleus by anchoring them to 14-3-3 protein and/or promoting their degradation in the cytoplasm. This interrupts their interactions with the TEAD family of transcription factors, which subsequently change the transcription of downstream genes. When the Hippo pathway is OFF, YAP/TAZ upstream kinases are inactivated, which results in translocation of YAP/TAZ into the nucleus to interact with TEAD transcription factor to activate downstream target genes.

Hippo pathway is a signal pathway that is involved in normal organ size control, tissue regeneration, angiogenesis, tumor suppression and metastasis, immune response, and drug resistance [1,2,3,4,5,6,7]. This evolutionary conserved signaling pathway was first identified in *Drosophila Melanogaster* and consists of a set of serine (S or Ser)/threonine (T or Thr) kinases [8]. The core components of the Hippo pathway in *Drosophila* includes two kinases Hippo (Hpo) and Warts (Wts), a scaffold protein Salvador (Sav), and an adaptor protein Mats [1,9]. These *Drosophila* Hippo core components have direct homologs in humans, including the Mammalian STE20-like 1/2 (MST1/2; Hpo), Large Tumor Suppressor 1/2 (LATS1/2; Wts), WW45 (Sav), and Mps One Binder 1 (MOB1; Mats) [10,11,12]. Following the activation of the Hippo pathway by cell-cell contact, extracellular matrix (ECM) stiffness, or cellular stress (e.g., DNA damage, nutrient deprivation, etc.), MST1/2 kinases are activated, which subsequently phosphorylate MOB1/2 adaptor protein and SAV1 scaffold protein that assist MST kinase to recruit and phosphorylate LATS1 at T1079 and LATS2 at T1041 [3,12,13]. SAV1 acts as a scaffold protein to bring LATS and MST together by binding to both of them [14]. MOB1 binds to LATS1/2 and induces the autophosphorylation of these kinases in their activation loop (S909 for LATS1 and S872 for LATS2), which in turn increases their kinase activity [15,16]. LATS1/2 can be also phosphorylated and activated by MAP4Ks, a member of mammalian Ste20-like family, which act in parallel to MST to enhance the activity of LATS1/2 kinases [17,18,19]. Activated LATS1/2 phosphorylate WW domain-containing transcription coactivators Yes-associated protein (YAP) at five sites (S61, S109, S127, S164, S381) and its paralog transcriptional coactivator with PDZ-binding Motif (TAZ)/WW Domain-Containing Transcription Regulator Protein 1 (WWTR1) at four sites (S66, S89, S117, S311) with a consensus phosphorylation motif of HxH/R/KxxS/T (H, histidine; R, arginine; K, lysine; x, any amino acid) [20,21,22,23,24]. Phosphorylation of S127 on YAP or S89 on TAZ by LATS creates a binding site for 14-3-3 protein that sequesters YAP/TAZ in the cytoplasm and inhibits YAP/TAZ activity [25,26] (Figure 1, “Hippo on”). Phosphorylation of S381 on YAP or S311 on TAZ by LATS can cause their sequential phosphorylation by CK1 kinase and subsequent degradation by SCF^β-TrCP^ E3 ligase [27,28]. On the other hand, inactivation of upstream core components of the Hippo pathway or dephosphorylation of 14-3-3 docking site on YAP or TAZ reduces YAP/TAZ phosphorylation and increases nuclear translocation of YAP/TAZ, thereby leading to YAP/TAZ activation (Figure 1, “Hippo OFF”). The activated YAP/TAZ can then bind to the TEAD family (TEAD1–4) of transcription factor which can activate the transcription of its downstream target genes (Figure 1, “Hippo OFF”) such as connective tissue growth factor (CTGF), cysteine-rich 61 (Cyr61), fibroblast growth factor (FGF1), AXL, BMP4, and PD-L1 [23,29,30,31,32,33]. These upregulated genes are involved in tumorigenesis including increased cell proliferation and cell migration, reduced cell death, and immune evasion. Therefore, altered expression or mutation in the Hippo pathway components can lead to abnormal cell growth, transformation, and tumor metastasis [34,35,36,37].

In addition to TEAD interaction, YAP/TAZ have also been demonstrated to regulate gene expression by forming a complex with SMADs [38,39], RUNX [40], TBX5 [41] and TP73 [42] transcription factors. Moreover, the Hippo pathway can crosstalk with a variety of signaling pathways such as WNT pathway [43,44], PI3K pathway [45,46,47], G protein-coupled receptors (GPCRs) [48] that can directly or indirectly regulate different components of the Hippo pathway. While a large numbers of Ser/Thr kinases (e.g., ILK, cyclin dependent kinase 1 (CDK1), Aurora A, NLK, PI3K, Protein kinase A (PKA)) [45,49,50,51,52,53,54,55,56,57,58,59,60] and tyrosine (Y or Tyr) kinases (e.g., c-ABL, SRC, VEGFR, AXL, FGFR, ERBB4) [53,61,62,63,64,65,66,67] have been reported to regulate the Hippo pathway, the regulatory mechanisms of phosphatases in this pathway are less understood. In this review, we describe the interaction of phosphatases and the Hippo pathway components and the underlying mechanism of Hippo regulation by phosphatases. 

### 1.2. Overview of Protein Phosphatases Family and Their Roles in Cancer

The reversible addition and removal of phosphate to proteins or lipids, phosphorylation, is a post-translational modification that is central for signal transduction in numerous vital cellular functions such as cell-cell communication, metabolism, cell proliferation and differentiation, apoptosis, and cell migration [68,69,70,71,72]. It is regulated by the opposing function of protein kinases (addition of phosphate) and phosphatases (removal of phosphate) [73]. It is shown that phosphorylation occurs in more than two-thirds of all cellular proteins encoded by the human genome [68]. Of all residue phosphorylation, phosphorylation of Ser, Thr, and Tyr accounts for approximately 87%, 12%, and 1%, respectively [74,75]. Phosphorylation on histidine and aspartate residues also sometimes occur but are rare and less stable when compared to Ser/Thr/Tyr phosphorylation [68]. Protein kinases are one of the largest gene family, making up about 2% of the human genome, whereas the number of protein phosphatases encoded is less than one-third of protein kinases [76,77]. In the past decades, most of the studies focus on the roles of kinases in cancer development and therapy due to the lack of new discoveries on phosphatases [78]. Targeting kinases with drugs has become one of the most promising cancer therapies [79]. However, mounting evidence suggests that phosphatases are also as important as kinases in many biological functions and diseases [80]. Therefore, the exploration of phosphatase function in normal physiology and pathophysiology is critical to provide very useful information for future targeting of phosphatases for cancer therapies. 

**Figure 2:**
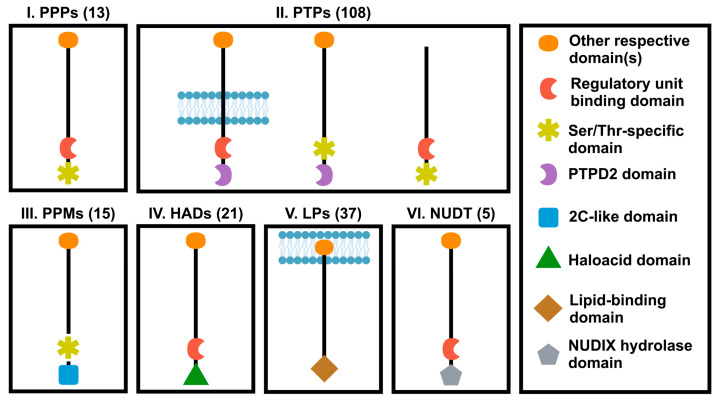
Classification of phosphatase superfamily. Protein phosphatases were classified into six different families according to the catalytic domains. PPP, phosphoprotein protein phosphatase family; PTPs, protein tyrosine phosphatase domain family; PPM, protein phosphatase 2C-like domain family; HAD, Haloacid dehalogenase-like hydrolase domain family; PPTD2, distal PTP domain; LPs, lipid domain family; NUDT, NUDIX hydrolase domain family. Numbers in parenthesis represent numbers of genes in each family.

Human genome encodes a total number of about 199 phosphatases that can recognize phospho-proteins or phospho-lipid and regulate their functions [77,81]. Protein phosphatases are divided into six groups based on their catalytic domain sequences of similarity (Figure 2): (1) Ser/Thr-specific protein phosphatases (PPP); (2) protein tyrosine phosphatases (PTPs); (3) protein phosphatase 2C-like domain containing metal-dependent protein phosphatases (PPMs); (4) Haloacid dehalogenase-like hydrolase (HAD) or HD domain phosphatases; (5) lipid phosphatases (LPs) including one of the following three subgroups: phosphatidic acid phosphatases, inositol monophosphatases and inositol polyphosphate-related phosphatase; (6) NUDIX hydrolase domain (NUDT) phosphatases. Each phosphatase group can be additionally subdivided into different classes [81].

Phosphatases are demonstrated to regulate the function of phosphorylated proteins by physical interaction with their substrates through three different ways [77]. The first mechanism relies on the presence of a targeting domain that binds to the catalytic domain of protein phosphatases by covalent bonds. For example, Src homology 2 (SH2) domain in SHP-2/protein tyrosine phosphatase non-receptor 11 (PTPN11) or FERM domain in PTPN21/PTPD1 binds to phosphorylated tyrosine (pY) of GAB1 or SRC kinases, respectively, which allows GAB1 or SRC expose to the catalytic domain of these phosphatases [82,83]. Complementary mechanism is the second type of interaction between phosphatases and their phosphorylated substrate. In this strategy, several small motifs in phosphatases directly interact with the substrate binding pocket of phospho-protein. For example, the proline (P)-rich motif of PTPN12/PTP-PEST docks into the SH3 domain of p130Cas in the substrate through complementary mechanism [77,84]. A similar type of interaction was also observed in a dual specificity phosphatase (DUSP) PYST1/MKP3 that targets and dephosphorylates ERKs, which in turn regulate MAPK pathway. Indeed, a motif includes 14 residues which is found in several PTPs such as PTP-SL and Striatal-enriched protein tyrosine phosphatase (STEP) as well as PYST1/MKP3 is important for the direct complex formation between these PTPs and ERK [85,86]. The third mechanism for the regulation of substrate by protein phosphatases is non-covalent binding of phosphatase and adaptor protein or regulatory subunits that are critical for interaction of phosphatases with their substrates. Protein dephosphorylation of ABL kinase by PTPN12/PTP-PEST tyrosine phosphatase, which is mediated by PST-PIP1 adaptor protein is considered as an example of this interaction strategy [87,88].

Since protein phosphorylation and dephosphorylation play crucial roles in the cell signaling processes, any alteration in the balance between phosphorylation and dephosphorylation level can result in various diseases including cancer [68,89,90]. As most kinases are demonstrated to play oncogenic role in cancer, and because of the opposing function of kinases and phosphatases, phosphatases are presumed as tumor suppressors [91]. However, in the last decades, a number of studies on the role of phosphatases demonstrated that the enzyme superfamily can be involved in either oncogenic or tumor suppressing processes [92]. For example, loss of many PTPs such as PTPN1/PTP1B, PTPN12/PTP-TEST and PTPN21 contributes to tumorigenesis or/and metastasis. On the other hand, PTP4A1, PTP4A3, PTPN1/PTP1B are overexpressed in various types of cancers [93]. 

## 2. Regulation of the Hippo Pathway by Phosphatases

### 2.1. Regulation of the Hippo Pathway by PSPs 

Protein Ser/Thr phosphatases (PSPs) are categorized into three different subfamilies according to their function and structure: PPPs, PPM, and aspartate-based phosphatases [94]. The PPPs family contains 13 members such as PP1, PP2A, PP2B, PP4, PP5, PP6 and PP7, which differ from each other based on their protein sequences and catalytic domain [95]. While PPPs have their catalytic domains interacting with regulatory subunits, the PPM family lacks the regulatory subunit and has several extra domains that play crucial roles in identification of specific substrates. Specifically, they comprise of enzymes which are dependent on Mg2+/Mn2+ such as PP2C and pyruvate dehydrogenase phosphatase [96]. As PPM differs from PPPs in their structure, PPPs inhibitors do not target the PPM family [97]. The third group of Aspartate based phosphatases are shown by F-cell production (FCP) and small carboxyl terminal domain (CTD) phosphatase (SCP) that dephosphorylate CTD of RNA polymerase II and SMAD 1, 2 and 3, respectively [96,97,98,99]. 

#### 2.1.1. PP2A and STRIPAK

Protein phosphatase type 2A (PP2A) comprise approximately 1% of total proteins in some mammalian tissues and together with protein phosphatase 1 (PP1) constitute more than 90% of the Ser/Thr phosphatase enzymes in most cells [94,100]. PP2A, as one of the most complex Ser/Thr phosphatases, was demonstrated to have promiscuous activity resulting in dephosphorylation of proteins involved in a diverse range of biological process such as cell cycle progression and cell death [101]. PP2A holoenzyme is a multi-subunit enzyme generated through interaction of a catalytic subunit (C), a regulatory subunit (B) and a scaffold subunit (A) that is responsible for bringing B and C subunits together [102]. It is demonstrated that alternative splicing generates a wide variety of B subunits that can be used in PP2A formation. Therefore, there is a broad array of PP2A holoenzymes that can recognize and act on a variety of phospho-Ser/Thr substrates [103,104]. In addition, B subunits can also function in a large complex. For example, among different types of B subunits, striatin family of proteins, including STRN1, STRN3, STRN4, have been identified as scaffold subunits in complex with other PP2A subunits and kinases to form a striatin-interacting phosphatase and kinase (STRIPAK) complex [105]. 

**Figure 3:**
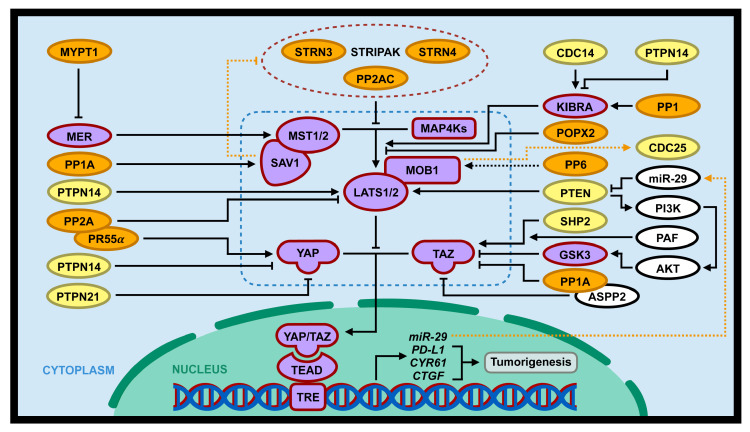
Interaction of the Hippo pathway and phosphatases in tumorigenesis. PSPs (PP2A/PR55α, PP1, PP1A, POPX2, and MYPT1; gene symbols with orange background) and PTPs (SHP2/PTPN1, PTPN14, PTPN21, CDC14, PTEN; gene symbols with yellow background) can regulate tumorigenesis through positive or negative regulation of the Hippo pathway (gene symbols with purple background). Dotted blue lines includes core components of the Hippo pathway (MST1/2, MAP4Ks, SAV1, MOB1, LATS1/2, and YAP/TAZ). In addition, the Hippo pathway can directly or indirectly regulate several phosphatases (PP2A, CDC25, and PTEN; dotted orange lines).

PP2A is deleted, deregulated and mutated in a wide variety of human cancers [106]. Recent studies revealed that PP2A-STRIPAK complex (PP2AC and STRN4) can induce tumorigenesis by activating YAP via suppression of MAP4K4/MST-LATS1/2 Hippo signaling pathway [107,108,109] (Figure 3; Table 1). It is also shown that there is some physical interaction between Hippo components (e.g., MAP4K, MST1/2, and NF2) and STRIPAK [107,109,110]. This STRIPAK-MST/MAP4K-MST-LATS-YAP signaling can also be activated by serum and lysophosphatidic acid stimulation [107]. As there are a variety of B subunits (regulatory subunit) in PP2A and this subunit determines specific enzyme function and substrate specificity, PP2A is involved in a wide range of cell processes and acts as either an oncogene or a tumor suppressor [111]. PR61 and PR72 regulatory subunits in PP2A are shown to act as tumor suppressors, while there are several subunits such as PR55α and PR130 that play roles as oncogenes [103,112,113,114]. A recent study reported that PP2A/PR55α can promote YAP oncoprotein activity [115]. Activation of YAP by PP2A/PR55α occurs in three different ways: (1) PP2A/PR55α directly dephosphorylate and activate YAP; (2) PP2A/ PR55α indirectly activate YAP by decreasing the stability of LATS kinase; (3) PP2A/PR55α activates YAP by inhibiting MOB-mediated LATS autophosphorylation (Figure 3). Altogether, this study revealed the crucial roles of PP2A/PR55α in activation of YAP in both human pancreatic normal and cancer cells [115]. 

In addition, there is another mechanism by which PP2A interacts with the Hippo pathway. Tang et al. showed for the first time that STRN3, as a regulatory subunit of PP2A in STRIPAK, can enhance PP2A-induced MST1/2 dephosphorylation and subsequently increase YAP activity (Figure 3). So, overexpression of STRN3 in gastric cancer cells lead to hyperactivation of YAP oncoprotein [105]. Many previous studies have demonstrated that MST1/2 and STRIPAK scaffolds are aberrantly expressed in different types of cancers [116,117,118,119], hence MST1/2 and STRN3 dysregulation may result in the development of various cancer. As it is shown that different regulatory subunits (B subunit) utilize different binding sites on PP2A, targeting the binding site for cancer treatment is a promising measure with less side effects. So, development of STRN3-derived Hippo-activating peptide (SHAP) as a specific inhibitor could block the complex formation of MST1/2 and STRN3, therefore preventing MST1/2 dephosphorylation and leading to Hippo reactivation [105]. 

#### 2.1.2. PP1 and PP1A

PP1 is a main Ser/Thr phosphatase involved in many significant cellular functions such as, apoptosis, transcription, protein synthesis, cell cycle and cell division [120]. This enzyme comprises one of the three conserved catalytic subunits, PP1A/ PPP1CA, PP1B/PPP1CB or PP1C/PPP1CC and a regulatory subunit such as PP1R3A and PP1R3B [121,122]. There are a diverse range of regulatory subunits which play crucial roles in targeting specific substrates by PP1. Therefore, the combination of different catalytic subunits and regulatory subunits is a determining factor for specific function of PP1 [123,124]. It has recently been shown that PP1 can regulate the Hippo pathway by directly binding and dephosphorylating KIBRA [125], a kidney and brain expressed protein that binds to and stimulates LATS1/2 kinase activity by modulating the hydrophobic motif site in LATS1/2 [126] (Figure 3; Table 1). KIBRA is a phosphoprotein that plays important roles in different signaling pathways including Hippo pathway in a phosphorylation-dependent manner [127,128]. As KIBRA is one of the substrates of PP1 and is dephosphorylated by this phosphatase [125], thereby PP1 may inhibit the Hippo pathway by suppressing LATS1/2 and subsequently activating YAP/TAZ activities via inhibition of KIBRA phosphorylation and activation (Figure 3; Table 1).

PP1A is one of the catalytic subunits of PP1 involved in a wide variety of cellular processes, such as cell proliferation, cell division and cell death [122,129,130]. This phosphatase has recently been shown to upregulate YAP transcriptional coactivating activity by directly dephosphorylating phosphorylated S127 (pS127) on YAP, resulting in enhanced YAP stability and YAP’s dissociation with 14-3-3 protein in the cytoplasm, consequently promoting YAP nuclear localization [20]. Consistently, Ser/Thr phosphatase inhibitor okadaic acid (OA) can enhance the levels of pS217-YAP by inhibiting PP1A-induced YAP dephosphorylation [131]. In addition to the dephosphorylation and activation of YAP, PP1A was demonstrated to form a complex with SAV1 and may regulate its activity by SAV1 dephosphorylation (Figure 3; Table 1) [132]. 

Moreover, PP1A has been shown to regulate YAP paralog TAZ. Since both PP1A and its interacting protein ASPP2 were detected to have physical interaction with TAZ, Liu et al. performed further experimentation to evaluate the effects of PP1A on TAZ activity [133]. Previous studies showed that TAZ phosphorylation at S89 and S311 results in cytoplasmic retention by binding to 14-3-3 protein and degradation by ubiquitination, respectively [28]. While PP1A dephosphorylates TAZ phospho-S89 (pS89) and phospho-S311 (pS89), cell treatment by PP1A inhibitor OA reverses PP1A’s effect on TAZ. In this way, PP1A promotes TAZ transcriptional co-activating activity and increase its nuclear accumulation (Figure 3; Table 1). In addition, PP1A-mediated TAZ dephosphorylation and activation depends on the presence of PY motif in ASPP2, a PP1A and TAZ binding protein [133]. Taken together, PP1A is involved in the regulation of the Hippo pathway by directly interacting with

#### 2.1.3. PP6

PP6 (PPP6C) is a highly conserved Ser/Thr phosphatase among eukaryotes and shares more than 50% identical amino acid sequence with PP4 and PP2A [134]. Similar to PP2A, PP6 is a holoenzyme with various regulatory subunits which is important in targeting specific substrate. It has recently been shown that the regulatory subunit of PP6 (e.g., PPP6R1–3) physically interacts with MOB1, a core component of the Hippo pathway, in a phosphorylation-dependent manner (Figure 3, Table 1) [110,135,136]. Upon phosphorylation, MOB1 binds to LATS to form an active complex which is able to phosphorylate and inactivate YAP/TAZ transcriptional coactivator [12]. Even though PP6 is likely to compete with MST1 for binding to and phosphorylation of MOB1, the exact molecular mechanism and functional significance underlying the regulation of the Hippo pathway via MOB1 by PP6 is unclear [110]. 

#### 2.1.4. POPX2

POPX2 (partner of PIX 2) is a Ser/Thr phosphatase that is part of PPM family. Recent studies also found that higher POPX2 expression is correlated with increased cancer cell motility and invasiveness in a variety of malignancies [137,138]. Four substrates have been reported susceptible to targeting by POPX2: p21-activated kinase (PAK), TGF-β activated kinase (TAK1), kinesin family member 3A (KIF3A) and calcium/calmodulin-dependent protein kinase II (CaMKII) [139,140,141,142]. Recent studies show that POPX2 also interacts with some Hippo pathway components such as MST1 and LATS1 [143]. Further experiments by phosphatase assay uncovered that POPX2 can dephosphorylate LATS at T1079. While LATS dephosphorylation at S909 by phosphatases such as PP1 was previously reported, there was no evidence of LATS1 dephosphorylation at T1079 [144]. Therefore, POPX2, as a LATS1 phosphatase, negatively regulates the tumor suppressor Hippo pathway through dephosphorylation and inactivation of LATS kinase, which in turn leads to the increased activation of YAP/TAZ oncogenes and its nuclear localization. YAP S127 phosphorylation by active form of LATS result in either YAP/TAZ cytoplasm sequestration or their degradation. So, loss of POPX2 was shown to be associated with decreased cellular amounts of YAP/TAZ, which is due to enhanced LATS kinase activity and increased phosphorylation and degradation of YAP [28,143].

#### 2.1.5. MYPT1

Myosin phosphatase targeting subunit 1 (MYPT1) is one of the subunits of myosin light chain phosphatase that is involved in biological processes including the cell cycle [145], development [146], cell-cell adhesion and cell movement [147] as well as regulation of smooth muscle contraction [148]. Interestingly, MYPT1 (PPP1R12A) is shown to form a complex with PP1, which subsequently promotes substrate specificity of MYPT1 [149,150]. Merlin/NF2, an upstream regulator of the Hippo pathway, is known as a substrate of the MYPT1-PP1 complex, which is dephosphorylated at S518 by the phosphatase function of the heterodimer, and in turn leads to its activation. Active form of Merlin/NF2 promotes the function of core kinase components of the Hippo pathway which subsequently leads to YAP/TAZ inhibition [151] and suppress cancer initiation and development [50]. Moreover, MYPT1 was down-regulated in ovarian cancer, which results in increased stemness through inactivation of NF2-MST1/2-LATS1/2-signaling and activation of YAP/TAZ activity [152]. Thus, MYPT1 is considered as one of the phosphatase regulators of Hippo signaling (Figure 3; Table 1).

### 2.2. Regulation of the Hippo Pathway by PTPs

The PTP superfamily is the most diverse phosphatase group including around 107 members. PTPs are divided into four separate subfamilies based on their catalytic domains and protein structure: class I cysteine-based PTPs, class II cysteine-based and tyrosine-specific, class III cysteine-based phosphatases, and aspartic acid-based PTPs [93,153,154]. Type I cysteine-based PTPs, which comprise 99 members, is the largest subfamily of PTPs that are subdivided into two distinct groups of classical PTPs and DUSPs [155]. Classical PTPs constitutes transmembrane, receptor-like PTPs (RPTPs) such as PTPR family members (e.g., PTPRA, PTPRB, and SAP1) and the intracellular, nonreceptor-like PTPs (NRPTPs) such as PTPN family members (e.g., PTP1B/PTPN1, SHP2/PTPN11, and PTPN14). DUSPs, which have both Ser/Thr and Tyr specific phosphatase activities, are divided into seven groups including mitogen-activated protein kinase phosphatases (MKPs), slingshot homolog (SSH) phosphatases, phosphatase of regenerating liver (PRL), myotubularin-related (MTMR) phosphatases, cell division cycle 14 (CDC14) phosphatase, PTEN phosphatase, and atypical DUSPs [156]. Class II cysteine-based PTPs only includes a single member named low-molecular-mass PTP (LMWPTP) with molecular weight of 18 kDa [157,158]. Class III cysteine-based phosphatases include three CDC members CDC25A, CDC25B and CDC25C that can activate CDKs by dephosphorylation of CDKs active site residue [158,159]. The last group of PTPs are aspartic acid based PTPs that use a different catalytic mechanism which comprises four members of EYA (eyes absent) and HAD (haloacid dehalogenase) [93,121,153,154,157]. 

#### 2.2.1. SHP2/PTPN11

SHP2 is a member of classical PTPs that is encoded by *PTPN11* and contains two SH2 domains in the N-terminal tail [160,161]. In the inactive state, the function of the PTP domain is suppressed by its intramolecular interaction with N-terminal SH2 domains. Upon targeting specific phosphotyrosine substrate, SH2 domains bind to the tyrosine residue and therefore the autoinhibition in SHP2 is abrogated, leading to the activation of enzyme function [161,162]. SHP2 has been shown to be regulated by hormones and growth factors, and is involved in a variety of cellular processes such as proliferation, differentiation and cell motility [163,164]. 

A recent study revealed that at low cell density, SHP2 is found to have physical interaction with Hippo pathway effectors YAP/TAZ (Figure 3; Table 1). Indeed, unphosphorylated YAP/TAZ enhance the accumulation of SHP2 in the nucleus through physical connection. In contrast at high cell density, SHP2 is entirely translocated to the cytoplasm where YAP/TAZ are sequestered when they are phosphorylated and inactivated [165,166]. Thus, due to complex formation of SHP2 with YAP/TAZ, cytoplasm accumulation of YAP/TAZ mediated by its phosphorylation can prevent nuclear function by sequestering SHP2 to the cytoplasm, while unphosphorylated YAP/TAZ in high cell density conditions mainly induce SHP2 nuclear accumulation. It is also interesting to note the complex between SHP2 and YAP/TAZ enhances the function of TEAD transcription factor and increase the transcription of its target genes [167].

Parafibromin (a nuclear scaffold protein) is a component in PAF (RNA polymerase II associated factor) complex, which plays a role in the regulation of gene transcription through interaction with transcriptional coactivators [168,169]. The interaction between YAP/TAZ and Parafibromin is shown to enhance their transcriptional activities. Upon dephosphorylation of tyrosine residues in Parafibromin by SHP2, the PAF complex can be recruited to the TEAD regulated target genes to stimulate the transcription of its downstream genes (Figure 3; Table 1) [167]. Inhibition of SHP2 lead to the decreased expression of the TEAD-YAP/TAZ mediated genes regulated such as *CTGF* and *CYR61*, while it does not have any effect on YAP expression levels [170].

#### 2.2.2. PTPN14

Protein tyrosine phosphatase non-receptor type 14 (PTPN14), a non-receptor tyrosine phosphatase, contains an N-terminal FERM domain, a C-terminal catalytic domain and two proline-rich motifs (PPxY), which are located in the central part of this phosphatase [53,171]. FERM domain is necessary for its interaction with cytoskeletal proteins and plasma membrane, while PPxY motifs facilitate the binding of PTPN14 to proteins containing WW domain, such as YAP and KIBRA [172]. There is some evidence indicating PTPN14 can interact with YAP and inhibit YAP co-transactivating activity and oncogenic function by increasing cytoplasmic sequestration of YAP [171,173,174]. It was also shown that PTPN14 interacts with WW domains of YAP via its PPxY motifs. However, there is controversy over the nature of the complex of YAP-PTPN14. While a study showed that one of two WW domains on YAP is crucial for its interaction with PTPN14 interaction, another study indicated that loss of both WW domains on YAP is important for disrupting its binding to PTPN14. In addition, Wang et al. showed that the second WW domain is more important than the first one in this interaction [174,175]. Surprisingly, inhibition of YAP by PTPN14 is independent of its phosphatase activity [174]. However, due to the presence of FERM domain, which facilitates the interaction of PTPN14 with plasma membrane and cytoskeleton, PTPN14 was also shown to be positioned in the adheren junction. There are some evidence indicating that PTPN14 is also localized in the nucleus and is associated with cell proliferation [173]. Huang et al. performed further study on the interaction between PTPN14 and YAP and found that PTPN14 does not have any effects on YAP phosphorylation at S127. Therefore, they suggested it is improbable that the interaction leads to YAP cytoplasmic retention, as the PTPN14/YAP complex may be positioned in the nucleus, as well. It is more likely PTPN14 can regulate YAP activity through competing with other YAP-interacting proteins for binding to WW domains and thereby negatively regulate YAP function [175].

Since previous studies clarified the role of PTPN14 as a negative regulator of YAP activity [171], it was interesting to shed light on the potential interaction between PTPN14 and other components of the Hippo pathway. In 2014, Wilson et al. found that PTPN14 can stimulate LATS activity not only through direct effects on the LATS kinase, but also through interaction with and regulation of KIBRA phosphoprotein (Figure 3; Table 1) [176]. Indeed, both PPxY motifs and PTP domain of PTPN14 are critical in forming a complex with WW domains of KIBRA [126]. A previous study demonstrated that LATS1 is more active when it is localized in the plasma membrane and thereby leads to increased LATS-dependent phosphorylation of YAP/TAZ [177]. As a result, through interaction with PTPN14, LATS1 kinase is localized to the plasma membrane which results in increased LATS1 kinase activity. However, different studies have uncovered that each of the three proteins PTPN14, KIBRA and LATS1 play separate roles in Hippo regulation as tumor-suppressors. They are also capable of inhibiting YAP activity more effectively through complex formation [176]. 

#### 2.2.3. PTPN21

Protein tyrosine phosphatase non-receptor type 21 (PTPN21) is involved in cellular processes by interacting with proto-oncogene tyrosine-protein kinase (SRC), focal adhesion kinase (FAK) and actin [178]. PTPN21 is also known as PTPD1 and includes a FERM domain in its N-terminal, which is associated with plasma membrane interactions and mediates the formation of a complex between PTPN21 and actin filaments [82,179]. Upon interaction with actin filaments, its catalytic domain, which is located in the PTPN21 C-terminal, is involved in actin-related cellular function such as cell migration and cell adhesion [178]. 

PTPN21 has recently been reported to be a novel upstream regulator of the Hippo pathway. Since PTPN21 is colocalized on the plasma membrane via its plasma membrane, interaction of PTPN21 with YAP inhibit YAP transcriptional co-activating and oncogenic activities by preventing YAP from translocating into the nucleus. Interestingly, PTPN21 only interacts with YAP but not its paralog TAZ [180]. PTPN21 and PTPN14 share similarities in their structure and both regulate the Hippo pathway through direct interaction with and suppression of YAP activity (Figure 3; Table 1). Therefore, knocking out either of them increased oncogenic activity of cancer cells by stimulation of YAP activity.

#### 2.2.4. CDC14A/B

CDC14 is a dual-specificity phosphatase involved in cell cycle regulation through inactivation of CDK and dephosphorylation of phosphorylated Ser/Thr, as well as tyrosine residues in CDK substrates [181]. In humans, there are three members of the conserved CDC14 including CDC14A, CDC14B and CDC14C [182]. Previous studies showed that KIBRA, as a Hippo pathway component, is phosphorylated mainly by Aurora kinases or CDK1 [125]. The phosphorylated KIBRA can then be dephosphorylated by CDC14. Aurora kinase rather than CDK1-mediated phosphorylation of KIBRA was proven to enhance LATS activity, which in turn induces YAP phosphorylation/inactivation (Figure 3; Table 1) [126]. Taken together, the CDC14-KIBRA-LATS1/2-YAP signaling pathway play important roles in the regulation of cell cycle progression and tumorigenesis. 

#### 2.2.5. Lipid Phosphatases PTEN

*Phosphatase and Tensin Homolog (PTEN*), which was first identified in 1997, is a PTP and tumor suppressor gene that is frequently mutated in a variety of human cancers [183,184,185]. It can dephosphorylate second messenger lipids including phosphatidylinositol-3, 4, 5-phosphate (PIP3) and phosphorylated proteins [186]. PTEN includes a catalytic domain positioned at the N-terminal, and C2 domain in the central region, and a flexible C-terminal regulatory region [187]. Recently, PTEN was shown to be connected to the Hippo pathway during tumorigenesis. Loss of *PTEN* was shown to promote cell proliferation and migration in vitro and tumor formation in mice of gastric cancer cells by abolishing the LATS-MOB1 interaction [188]. In addition, loss of *PTEN* function by mutations activates PI3K-AKT signaling, which subsequently enhances TAZ levels through inhibition of its inhibitor GSK3 [189] (Figure 3; Table 1). 

## 3. Regulation of Phosphatases by the Hippo Pathway 

### 3.1. PP2A

It has been shown that the N-terminal phosphatase-inhibitory domain (PID) of Hippo core component scaffold protein SAV1 can directly interact with the catalytic domain of PP2A in STRIPAK and suppress phosphatase activity. This consequently enhances the activity of MST1/2 by preventing MST1/2 phosphorylation loop from dephosphorylation by STRIPAK (Figure 3; Table 1) [190,191].

### 3.2. CDC25B

CDC25 phosphatase family is a protein phosphatase which plays an important role in the regulation of the cell cycle [192]. The dual specificity protein phosphatases can activate the function of CDK1 or CDK2 by dephosphorylation of two residues (Thr14 and Tyr15) and promote transition of cell-cycle to the subsequent phase. In humans, there are three isoforms in the CDC25 family including CDC25A, CDC25B and CDC25C [193]. CDC25B isoform, which is found in both the nucleus and centrosome, is shown in a complex with LATS1. While CDC25B is not a direct substrate of LATS1 kinase, inactivation of LATS1 causes abnormal accumulation of CDC25B, which leads to hyperactivation of its substrate CDK2, resulting in increased centrosome duplication. LATS2 is also shown to bind to CDC25B with less intensity [194]. The N-terminal fragment of LATS1 including the UBA (ubiquitin-associated) domain is critical for the interaction with CDC25B. UBA can ease recruitment of ubiquitinated proteins to the proteasome and facilitate its degradation, thereby it is probable that the UBA domain located in LATS1 can form a complex with ubiquitinated CDC25B and mediate proteasome degradation. Importantly, in a variety of cancers, high expression levels of CDC25B were related with loss of function of LATS1/2 or mutation in N-terminal region of LATS1/2 [194]. Moreover, Gerlach et al. discovered that *Drosophila* homolog YAP/TAZ, yki, can promote cytokinesis failure by transcriptionally upregulating *string*, a *Drosophila* homolog of CDC25 [195]. Cytokinesis failure (CF) leads to the production of polyploid cells, and may be considered as a cause of tumor formation [196]. In fact, increased expression of *string* in cells with CF in *Drosophila* leads to oncogenic transformation, which is triggered by stimulation of cell cycle progression. Taken together, inactivation of human LATS1/2 or activation of YAP/TAZ/Yki may lead to the upregulation of CDC25/*string*, which results in genetic instability such as centrosome amplification and CF, and eventually, human malignancies (Figure 3; Table 1). 

### 3.3. PTEN

While PTEN regulates Hippo pathway through different mechanisms mentioned above, there is some evidence demonstrating that PTEN can also be regulated through components of the Hippo pathway. It is shown that knockout of YAP1 leads to PTEN upregulation, which subsequently reduces phosphorylation levels of AKT and attenuates its activity. PI3K/AKT controls various biological functions such as cell proliferation, cell growth and cell death [197]. In addition, overexpression of YAP oncoprotein is correlated with reduced expression levels of PTEN and thereby elevated phosphorylation of AKT, which leads to increased breast cancer cell proliferation (Figure 3; Table 1) [198]. Moreover, overexpression of YAP is also shown to cause increased cell size, tissue growth, and hyperplasia through a miR-29-PTEN-PI3K-AKT signaling pathway [199] (Figure 3; Table 1).

## 4. Ongoing Challenges

It is clear that the dysregulated Hippo tumor suppressor pathway is associated with cancer initiation and development, and therefore evaluation of its major regulators is considered as a critical step in cancer treatment. Although phosphatases are implicated to regulate the Hippo pathway, there are still some challenges in the field that need to be taken into consideration. Some phosphatases, such as PP2A, target numerous substrates which may be involved in not only the Hippo pathway, but also in other signaling pathways [90,101,200]. Therefore, inhibition of the oncogenic phosphatases may have an impact on tumorigenesis through Hippo-dependent and Hippo-independent pathways. While the interaction between PP2A and Hippo components induces oncogenic transformation, which is mediated by dephosphorylation and inactivation of the Ser/Thr kinases via PP2A [115], the PP2A function is suppressed in different types of cancers. The phosphatases play its tumor suppressor roles through inhibition of mitogenic signals, suppression of cell cycle transition and inhibition of the Wnt signaling pathway [201,202]. Therefore, some phosphatases act as both oncogenes and tumor suppressors, thus adding complexity to inhibitor usage in cancer treatment. Therefore, more experimentation is required to evaluate the sensitivity of the inhibitors by disrupting the interaction of the phosphatases with Hippo components without any effects on the role of phosphatases on other pathways. Some investigation on the binding site of the phosphatases and the Hippo components may be helpful to design specific inhibitors, which can effectively influence the Hippo pathway exclusively apart from other signaling pathways. Another challenge related to the phosphatases regulating the Hippo pathway is OA inhibitor usage, which suppresses several phosphatases such as PP6 and PP2A. While researchers are evaluating the roles of PP2A in the Hippo pathway by OA-mediated PP2A inhibition, some of the phenotypes induced by the inhibitor may also be associated with PP1A, as the inhibitor is indiscriminate of the two phosphatases [203].

As discussed above, some phosphatases regulate the Hippo pathway through regulation of YAP oncogenic function. It is also well demonstrated that high cell density situations enhance YAP phosphorylation, so there is a need for some studies to clarify whether the effects of the phosphatases on the Hippo pathway is dependent on cell-density [20].

## 5. Conclusions and Future Directions

Balanced regulation of protein phosphorylation by kinases and phosphatases is critical in almost all biological processes. Although dysregulation of protein phosphorylation caused by activation or inactivation of kinases or phosphatases contributes to various human diseases including cancer, compared to kinases, the molecular mechanisms of phosphatases are relatively less known. In the last decade, many studies strongly suggest that several phosphatases (e.g., PP2A, PP1, STRIPAK, PTPN14, etc.) can regulate cancer cell proliferation, cell migration, and genetic instability through interaction with the Hippo pathway. However, there are over 199 phosphatases in the human genome. It remains unclear how other phosphatases interact with the Hippo pathway in many other biological processes. In addition, although many drugs targeting oncogenic kinases have been used for the treatment of cancers, only a few drugs targeting PTPs (e.g., PTP1B, SHP2, CDC25, and PRLs) are under clinical trials [204]. Given the roles of Hippo in tumorigenesis, angiogenesis, metastasis, drug resistance, and immune response [2,5,6,7,37], further elucidation of novel interactions between phosphatases and the Hippo pathway will provide very useful information for the targeting of phosphatases alone or in combination with Hippo-targeted drugs for the effective treatment of drug-resistant or metastasis cancers in the future. 

## Figures and Tables

**Table 1:** Interaction of phosphatase and the Hippo pathway.

Family	Phosphatase	Mechanism	References
*Regulation of the Hippo pathway by phosphatase*			
PSPs	PP2A-STRIPAK complex	Activates YAP by suppressing MAP4K4/MST-LATS1/2 or enhancing MST1/2 dephosphorylation	[110,111,112]
	PP2A/PR55α	Promotes YAP activity by dephosphorylating YAP, decreasing LAST stability, and inhibiting MOB-mediated LATS autophosphorylation	[118]
	PP1/PP1A	(1) PP1 suppresses LATS1/2 by dephosphorylating LATS activator KIBRA; PP1 dephosphorylates TAZ and increases its transcriptional coactivity	[128,136]
		(2) PP1A can directly dephosphorylate YAP; forms a complex with SAV1	[20,135]
	PP6	Physically interacts with MOB1	[138,139]
	POPX2	Inhibits LATS activity by dephosphorylating LATS	[146,147]
	MYPT1	Activates Merlin by dephosphorylates it at S518	[153,154]
PTPs	SHP2/PTPN11	Physically interacts with YAP/TAZ and activates YAP/TAZ-TEAD transactivating activity	[169]
	PTPN14	(1) Inhibits YAP co-transactivating activity and oncogenic function by increasing cytoplasmic sequestration of YAP in the cytoplasm;	[173,174,175,176]
		(2) Stimulate LATS activity by increasing its plasma membrane localization or regulating KIBRA	[13,178]
	PTPN21	Inhibits YAP transcriptional co-activating activity and oncogenic function by preventing YAP from translocating into the nucleus	[181]
	CDC14A/B	Dephosphorylates KIBRA	[125]
	PTEN	Loss of PTEN abolishes the LATS-MOB1 interaction and promote tumorigenesis, and activates PI3K-AKT signaling, which subsequently enhances TAZ levels	[189,190]
*Regulation of phosphatases by the Hippo pathway*			
PSPs	PP2A	SAV1 can directly interact with PP2A in STRIPAK and suppress its phosphatase activity	[191,192]
PTPs	CDC25B	Inactivation of LATS1 causes abnormal accumulation of CDC25B; Yki promotes cytokinesis failure by transcriptionally upregulating *string,* a *Drosophila* homolog of CDC25	[195,196]
	PTEN	Knockout of YAP1 leads to PTEN upregulation; overexpression of YAP is correlated with reduced expression levels of PTEN	[198,199]

Three components of the Hippo pathway, SAV, TAZ and YAP (Figure 3; Table 1).
